# Human immunodeficiency virus infection and cardiovascular risk

**DOI:** 10.1186/1471-2334-14-S7-P83

**Published:** 2014-10-15

**Authors:** Erzsébet Iringó Zaharia-Kézdi, Carmen Chiriac, Andrea Incze, Nina Șincu, Előd Nagy, Ildikó Dósa

**Affiliations:** 1 Clinic of Infectious Diseases I, University of Medicine and Pharmacy Tîrgu Mureş, Romania; 2 County Hospital Laboratory, University of Medicine and Pharmacy Tîrgu Mureş, Romania; 3 University of Medicine and Pharmacy Tîrgu Mureş, Romania

## Background

Cardiovascular diseases are the second leading non-AIDS dependent cause of mortality for human immunodeficiency virus-infected (HIV) patients. Thus, the evaluation of cardiovascular risk factors (CVRF) and the identification of persons at risk are necessary for the implementation of prevention methods.

## Methods

A retrospective cross-sectional study was performed on a group of 50 HIV patients at the I Clinic of Infectious Diseases Tg Mureş (33 male, average age: 27.1±7; average duration of antiretroviral treatment: 11.16±6.88 years; average TCD4+ lymphocyte (LTCD4+) count on last evaluation: 593.98±392.55 cells/µL; 38 in AIDS stage). From the moment of diagnosis until July 2014, several classic CVRF have been studied: age, sex, smoking, obesity, dyslipidemia, diabetes, hypertension and global cardiovascular risk (CVR) estimated with Framingham score. The results were correlated with: antiretroviral treatment (ART), the number of ART combinations, LTCD4+ and hepatitis B (HBV) coinfection. For statistical analysis the Student and Mann-Whitney tests were used with 95% confidence interval and statistical significance p<0.05.

## Results

CVRF: hypertriglyceridemia, 29 patients (22 in stage C), decreased high-density lipoproteins (HDL) 23 (elevated, 10), hypercholesterolemia 12, elevated low-density lipoprotein (LDL) 7, smoking habit 16, obese 3, overweight 5, underweight 8, hypertension 2, diabetes 1. The Framingham test global scores: 6 moderate, 1 elevated, 43 decreased. A significant statistical difference can be observed between the initial and final values of: total cholesterol (TC), LDL and triglycerides (TG) p=0.0002, p=0.0495, p=0.0002. Patients with ≥3 combinations of ART had a significantly larger average value of TC, LDL and TG (p=0.030, p=0.041, p=0.027) compared to those with 1 combination.

Significant statistical differences are registered between: average TC and TG values (p=0.0001 p=0.0013) depending on the treatment with or without protease inhibitors (PI); between HDL values for patients with LTCD4+ ≥500 cells/µL and those with TCD4+ <500 cells/µL (p=0.028). Average TG values were significantly higher for HBV coinfections (p=0.0349).

## Conclusion

The most frequent CVRF were: hypertriglyceridemia (58%), fall of HDL levels (46%), smoking (32%), hypercholesterolemia (24%), decreased LDL levels (14%). The lipid levels are influenced by: type of ART, number of combinations, presence of HBV and the advanced stage of disease. High levels of LTCD4+ and HDL can be considered protective factors. Because of the existence of these risks, the initiation of a program for the reduction of cardiovascular risks is necessary.

